# Research on Rare Diseases in Germany – The cancer predisposition syndrome registry

**DOI:** 10.25646/11828

**Published:** 2023-12-13

**Authors:** Christina M. Dutzmann, Nathalie E. Palmaers, Lucas J. Müntnich, Farina J. Strüwe, Judith Penkert, Birte Sänger, Beatrice Hoffmann, Anja Karow, Christina Reimer, Tanja Gerasimov, Marena R. Niewisch, Christian P. Kratz

**Affiliations:** Department of Pediatric Hematology and Oncology, Hannover Medical School

**Keywords:** CANCER PREDISPOSITION, PREVENTION, SURVEILLANCE, LI-FRAUMENI SYNDROME, RARE DISEASES, CHILDREN

## Abstract

**Background:**

Cancer predisposition syndromes (CPS) are rare diseases that are associated with an increased risk of cancer due to genetic alterations. At least 8 % of all cases of childhood cancer are attributable to CPS [1, 2]. The CPS registry was launched in 2017 to learn more about CPS and to improve the care to those afflicted by these diseases.

**Methods:**

This is an internationally networked registry with associated accompanying studies that investigate cancer risks and spectra, the possibilities of cancer prevention, early detection and therapy.

**Results:**

For several of these syndromes, new insights into the cancer risks and cancer types as well as factors modifying cancer risk have been gained. In addition, experimental, psycho-oncological, preclinical and clinical studies were initiated.

**Conclusions:**

The CPS registry is an example of how progress can be made within a short period of time to the benefit of individuals with rare diseases through systematic data collection and research.

## Introduction

Cancer predisposition syndromes (CPS) are rare genetic diseases that are associated with an increased risk of cancer compared to healthy individuals. In both children and adults, CPS are among the most important risk factors for cancer and at least 8 % [1, 2] of people with cancer suffer from a CPS. One of the better-known CPS is Li-Fraumeni Syndrome (LFS) with 16,000 afflicted individuals in Germany with a prevalence of 1:5,000 [3]. LFS is associated with a drastically increased risk of a variety of cancers in both childhood and adulthood. Various questions arise for each of the many different CPS: How high is the cancer risk in different phases of life? What types of cancer occur? Which genetic and environmental factors influence the cancer risk of the afflicted? How can the cancer risk of the afflicted be reduced? How can cancer be recognised early in the afflicted? What are the special features of cancer treatment? What are the psycho-oncological consequences and needs?

In order to address these questions, the CPS registry (www.krebs-praedisposition.de) was started in 2017. Research for people with CPS benefits not only people with CPS, but also individuals with sporadic, i.e.non-hereditary, types of cancer, as hereditary and sporadic cancers are based on identical signalling pathways. For example, Li-Fraumeni Syndrome is caused by hereditary variants in *TP53*, a gene that is also altered in many sporadic types of cancer. CPS research is therefore of great importance for cancer research in general.

**Infobox Translational Research on Rare Diseases – a funding priority of the Federal Ministry of Education and Research**

A disease is considered rare if fewer than five in 10,000 people are affected by such a diagnosis. More than 8,000 rare diseases are known. It is estimated that more than four million people in Germany alone are affected by a rare disease.

Around 80 % of rare diseases are genetically determined, some diseases cause their first symptoms in childhood. However, the causes of the disease are often unexplored. The relatively small number of people affected, experts and suitable medicines complicate the path to a diagnosis and appropriate therapy. If there is no diagnosis or it can only be made at a late stage, irreversible courses of the disease are often a result. Therefore, research is vital for those affected. Basic research plays an important role here: it not only provides new insights into rare diseases, but can also contribute to a better understanding of more common diseases.

Since 2003, the Federal Ministry of Education and Research (BMBF) has been funding networks that jointly research causes and therapeutic approaches for rare diseases at various university locations. A coordination office supports these networks, among other things, in presenting their results to the public (see also https://www.research4rare.de/wp-content/uploads/2023/05/Poster_R4R_engl_2019-2026.pdf). At the European level, the research consortia are involved in the European Reference Networks (ERN) on rare diseases. In addition, there are international programmes, such as the European Joint Programme on Rare Diseases (EJP RD) for research into the diagnosis and therapy of rare diseases, in which the BMBF also participates.

## Project

The CPS registry was opened in 2017 as a non-interventional observational study with the primary aim of addressing the questions mentioned above. The focus of data collection is on genetic findings, detailed information on cancer in patients and their relatives as well as early detection measures. A biobank and an MRI image database are an integral part of the registry. The CPS registry serves as the basis for the ADDRess (Translational Research for Persons with Abnormal DNA Damage Response) research network, which focuses explicitly on the CPS subgroup of DNA repair defects associated with a particularly high cancer risk. This national consortium addresses various issues relating to the causes of CPS, psycho-oncology, surveillance by MRI and/or liquid biopsy (detection of cell-free tumour DNA via a blood sample), mechanisms of carcinogenesis and novel treatments.

The aim of the registry is to create research structures, particularly for patients, which are not covered by the existing activities of other research groups, such as in the areas of familial breast and ovarian cancer and hereditary colon cancer. There is also a close exchange with the established therapy studies in the field of paediatric oncology. The registry is open to adults, children and adolescents. The aim is therefore to permanently obtain findings on various CPS that are relevant in adulthood, childhood and adolescence.

## Results and classification

**Figure 1:**
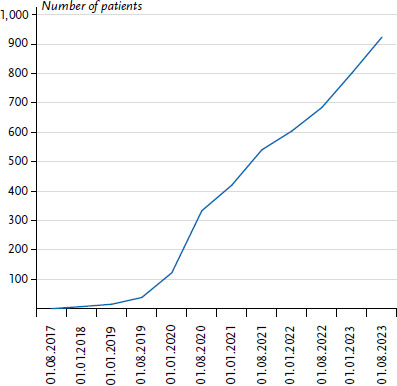
Cumulative registrations in the CPS registry Source: CPS Registry, as of: 31 July 2023

After the registry was opened in 2017 in collaboration with the Department of Paediatric Haematology and Oncology of Hannover Medical School and the Hopp Children's Cancer Center Heidelberg, 923 patients were registered by July 2023 (Figure 1). Each year, there are some 150 new entries, with an upward trend. The registry is open to patients of all ages and with all types of CPS. Inclusion in the registry is possible either via an attending centre, which is the case especially in the field of paediatric oncology, or by the patients themselves as self-registration by contacting the CPS registry directly and can be done by both German and international patients. Furthermore, an independent CPS outpatient clinic has been established at the Department of Paediatric Haematology and Oncology at Hannover Medical School, where patients with CPS can receive counselling and treatment. It is also possible to carry out early cancer detection screening there, especially for people with LFS and for children and adolescents with diverse CPS. The close spatial, personnel and organisational cooperation between the CPS outpatient clinic and the CPS registry enables not only the recruitment of numerous patients for the registry, but also enhances the expertise of the attending physicians based on the knowledge gained from the registry activities. The increasing registration numbers (Figure 1) offer the opportunity to carry out meaningful analyses of the data and to better understand various types of CPS. Individuals afflicted by various CPS can benefit directly from these insights and their implementation in clinical settings, for example by adapting the early detection of cancer. Inclusion in the CPS register is by no means a prerequisite for connection to the CPS outpatient clinic, though. In addition, afflicted individuals often become aware of the activities of the CPS register via social networks and patient organisations. In addition, the website www.krebs-praedisposition.de has been set up, which offers a wealth of information on many different CPS for both healthcare professionals and patients in addition to the online representation of the CPS Registry. In addition to data collection, professional, individualised counselling for patients with CPS is another important aspect of the work in the registry. This takes place either by telephone via the CPS registry physicians or during a personal appointment by the CPS outpatient physicians in the above-mentioned CPS outpatient clinic at Hannover Medical School, where appointments for patients from all over Germany are made. Through its sub-projects and close cooperation with the CPS outpatient clinic, the registry combines research, clinical counselling and early cancer detection, so that the knowledge thus gained can lead to significant improvements in the care provided to people with CPS.

The results obtained to date include two population-based epidemiological studies in which the risk of cancer in childhood was calculated for various CPS [4, 5]. This was done for patients with Fanconi anaemia (FA), ataxia teleangiectasia (AT) and the Beckwith-Wiedemann spectrum (BWSp) through a comparison to data from the German Childhood Cancer Registry, in which almost all cases of childhood cancer are registered. The risk of developing cancer by the age of 18 was found to be 39 times higher in children with FA and 56 times higher in children with AT (as compared to the general population of the same age). Children with BWSp had a 32-fold increased risk of developing cancer by the age of 15. In addition, the cancer spectrum for the aforementioned diseases and subgroups thereof in childhood was determined. This work has important implications for risk-adapted early detection, which are already being implemented clinically. For example, certain examinations may be started at a later age or early detection measures may be intensified or relaxed for certain subgroups depending on the cancer risk. Specifically, this means, e.g., that the annual bone marrow puncture as part of early cancer detection in children with FA subgroups FA-A, FA-C or FA-G is not started until after the age of three, as the earliest occurrence of myelodysplastic syndrome or acute myeloid leukaemia was not observed at the age of four. In contrast, more comprehensive cancer screening is recommended for children with FA subgroups FA-D1 and FA-N as the cancer spectrum is different. An increased risk of cancer, particularly hepatoblastoma and nephroblastoma, has been observed in children with BWSp, which supports the recommended regular sonographic and clinical examinations. Patients with a ‘loss of methylation in imprinting centre 2 (IC2-LOM)’ are an exception, as these early detection examinations can be dispensed in this cohort due to the low risk of cancer.

Other studies [6, 7] looked at the spectrum of diseases associated with germline variants in *TP53* or Li-Fraumeni Syndrome (LFS), one of the most important CPS. It was shown that different clinical manifestations and courses can occur within LFS. The spectrum was used to reveal clinically relevant genotype-phenotype correlations, showing that different genetic changes within a gene can, for example, lead to different types of cancer and different ages at onset. This may be important for risk-adapted early detection in the future.

Another study quantified the significance of adult CPS genes in childhood and adolescence for the first time [8]. Adult CPS genes are known to lead to an increased risk of cancer in adulthood. Examples include the *BRCA_1_* and *BRCA_2_* genes, which are associated with an increased risk of breast cancer. Variants in these genes already play a role in childhood and adolescence, but with low penetrance (the penetrance describes the extent to which a genetic change is clinically expressed). This work has important implications for genetic counselling, as such variants are increasingly being identified by panel sequencing, a method of comprehensive human genetic testing. Cancer screening is currently not recommended for healthy children and adolescents in whom one of these variants has been detected. However, the results of further studies showed that children and adolescents who have already experienced cancer and who have a pathogenic variant in an adult CPS gene may be at increased risk of developing a second cancer (second neoplasia), especially if they have undergone genotoxic therapy such as radiotherapy.

The CPS registry has already provided valuable insights for the provision of care to people with CPS. By continuing the CPS registry and the already established sub-projects ADDRess and Liquid Biopsy, further significant research results are to be obtained that will improve the early detection of cancer in people with CPS and optimise psychosocial support for afflicted patients and their families. In addition, further causes of CPS are to be identified and novel therapeutic approaches are to be generated. One important aspect of the CPS registry in conjunction with the CPS outpatient clinic is to offer patients a point of contact at which their rare disease is taken seriously and where they can get expert advice and optimal treatment.

## Key statement

- At least 8 % of all cases of cancer are due to a cancer predisposition syndrome.
- Cancer predisposition syndromes are therefore among the most important cancer risk factors in children and adults alike.
- Research into cancer predisposition syndromes is essential in order to optimise the care provided to the afflicted.
